# Editorial: Biotechnological and genomic approaches for enhancing agronomic performance of crops

**DOI:** 10.3389/fgene.2022.991630

**Published:** 2022-10-04

**Authors:** Reyazul R. Mir, Himabindu Kudapa, Sreepriya Pramod, Ramsey S. Lewis

**Affiliations:** ^1^ Division of Genetics and Plant Breeding, Faculty of Agriculture, Sher-e-Kashmir University of Agricultural Sciences and Technology Srinagar, Srinagar, India; ^2^ International Crops Research Institute for the Semi-Arid Tropics (ICRISAT), Patancheru, India; ^3^ Altria Client Services LLC, Center for Research and Technology, Richmond, VA, United States; ^4^ Department of Crop and Soil Sciences, North Carolina State University, Raleigh, NC, United States

**Keywords:** biotechnology and bioinformatics, genomics (genetic tests and databases), crop improvement, gene, markers

## Why do we need to care and what is the solution?

The knowledge of plant genetics and plant genomics is very important in advancing our understanding of the fundamental processes in the science of plant biology. Different biotic and abiotic stresses coupled with changing climatic conditions cause economic losses in most major crops worldwide. Pioneering strategies for improving plant health and defense mechanisms can inform vital priorities for maintaining agricultural production and supply a rapidly expanding global population. Despite the advances made in plant breeding, there is a need to support our cultivar development programs to understand the limitations in productivity and to direct efforts towards gaining precise understanding of the target genes responsible for controlling the traits of interest in any given crop. The advent of advanced genomic tools and technologies can now enable developing high-yield, stress-tolerant crop varieties (*see* Raza et al., 2021; Pazhamala et al., 2021).

The science of plant breeding is most important and helps in delivering new varieties with novel trait combinations and improved agronomic performance. The science of plant breeding is continuously evolving and one of the reasons for this continual progress is the integration of biotechnological/genomics tools and techniques with plant breeding. Great success has been achieved over the years in trait phenotyping, genotyping approaches/methods, and biotechnological approaches and their use in crop improvement programs (Mir et al., 2015, 2019). The progress and new advancements made in these research areas continue to reduce the time and cost from discovery of genes/quantitative trait loci (QTLs) for traits of interest followed by their deployment in existing improved cultivars through marker-assisted selection (MAS). The broader research community has provided a wealth of genomics/transcriptomics resources, as evident by the fact that an increasing number of high-quality reference or draft genomes, pan-genomes, transcriptomes, and molecular markers are becoming available for almost all crop plants.

A variety of different kinds of molecular markers have been developed in almost all important crop plants in the world (Gupta et al., 2008, 2013a; Mir et al., 2013a,b; Mir and Varshney 2013; Tyagi et al., 2019, 2021; Kumar et al., 2021; Sagwal et al., 2022). More new types of molecular markers have been discovered and used for crop improvement programs. For instance, molecular markers have been used extensively in different activities including the study of genetic diversity of different crop germplasm resources (Banerjee et al., 2012; Choudhary et al., 2021; Shafi et al., 2021; Tahir et al., 2021), study of population structure, development of high-density genetic/linkage maps, and mapping genes through traditional quantitative trait loci (QTL) mapping/family mapping and somewhat modern genome-wide association studies (GWAS)/population mapping (Gupta et al., 2013b). In addition, these markers have been also used extensively in the development of improved crop varieties through modern breeding approaches like marker-assisted selection (MAS)/marker-assisted gene pyramiding, marker-assisted recurrent selection (MARS), and genomic selection (GS)/genome-wide selection (GWS) (Mir et al., 2012). For example, in rice through marker-assisted gene pyramiding two major bacterial blight (*Xa21* and *xa13*) and two major blast resistance genes (*Pi54* and *Pi1*) were introgressed into a mega variety Tellahamsa (Jamaloddin et al., 2020).

Newer emerging molecular markers, also known as next-generation molecular markers are expected to accelerate crop improvement programs (Mir et al., 2022). For instance, new markers including structural variations (SVs) and k-mers are believed to be extremely useful in GWAS and in other gene discovery programs (Mir et al., 2022). New genomics tools are continuously evolving. For instance, whole-genome re-sequencing (WGRS), QTL sequencing (QTL-Seq) and restriction site-associated DNA (RAD) genotyping have recently emerged and have revolutionized the gene discovery programs and therefore crop improvement programs (Mir et al., 2013a; Mir and Varshney 2013; Mir et al., 2022). The first set of tools for fine mapping of genes/QTLs influencing economically significant quantitative traits, such as biotic/abiotic stressors and agricultural yields, are reference genomes, transcriptomes, and molecular markers (Barmukh et al., 2022).

The study of genetics and development of improved varieties is quite easy for traits controlled by one of few genes (Qualitative traits). However, for quantitative traits, which are controlled by large number of minor genes, the improvement is not straightforward. For these quantitative traits, some sophisticated approaches have been developed for mapping genes/QTLs responsible for these traits followed by their deployment into cultivars. Several genome mapping approaches have been used in crop plants for decades (Mir et al., 2012; Gupta et al., 2013b). Among these approaches, QTL mapping is one of the most important traditional mapping approaches for mapping genes affecting quantitative traits. The QTL mapping method involves the use of bi-parental mapping populations developed using contrasting parental genotypes. Although used in many crop plants for genetic dissection, the QTL mapping method has several limitations and some of them are as follows: 1) involves the development of bi-parental mapping populations that can be time-intensive; 2) populations are derived using only two parental genotypes resulting in low mapping resolution and little diversity; 3) low rates of marker polymorphism. On the other hand, alternative approaches now widely used in genome mapping projects are genome-wide association studies (GWAS) or association mapping analyses. These methods have several advantages over traditional QTL mapping approaches. Some of these advantages are enumerated below: 1) diverse unrelated genotypes are used; 2) high mapping resolution due to a several cycles of historical recombination events; (iii) no marker is mono-morphic; and 4) there is no requirement to develop mapping populations. Additionally, several new advances such as gene-based trait mapping have aided gene discovery in crop plants. The increased speed of rapid sequencing has resulted in a decline in sequencing costs. These advancements facilitated the generation of draft genomes of >800 plant species, and the number continues to increase (Kulwal et al., 2022). The sequencing of a large number of plant genomes have not only facilitated the discovery of genes, alleles, markers, *etc.*, but also introduced the new concept of the plant pangenome, consisting of the “core genome” and the “dispensable genome” (*see* Kahn et al., 2020). Once identified, desirable allelic variability can be utilized in breeding programs through marker-assisted selection (MAS) including marker-assisted gene pyramiding. These breeding approaches rely on the use of markers associated with the QTL/gene of interest for foreground selection in segregating populations over the generations. Furthermore, whole genome screening combined with the use of novel breeding technologies, such as gene editing, enables more precise trait modification. The successful transfer of cultivars with improved agronomic performance from research settings to commercial growing situations continues to be the ultimate goal.

This Research Topic on ‘*Biotechnological and genomic approaches for enhancing agronomic performance of crops*’ contributes to different aspects of plants genetics, genomics and biology. The Research Topic includes a total of 18 articles (fifteen research articles and three review articles) that cover recent advances in plant/crop research in the context of genomic resource development in different crops, functional genomics, gene discovery through different approaches, and understanding mechanisms of tolerance to different biotic/abiotic stresses. Several omics tools and technologies have been used to mine/characterize genes and gene families to facilitate their use in crop improvement. Initial characterization of genes/gene families has been limited to model plants - *Arabidopsis thaliana*, *Brachypodium distachyon*, *Medicago truncatula*, due to their typical features such as small genome size, ease of cultivation, dwarf plant phenotype and responsiveness to genetic transformation.

Next generation sequencing (NGS) has enabled the generation of comprehensive genomic and transcriptomic resources in many crops including model cereal (rice [*Oryza sativa*], wheat [*Triticum aestivum*], maize [*Zea mays*].) and legume (common bean [*Phaseolus vulgaris*], pea [*Pisum sativum*], soybean [*Glycine max*], chickpea [*Cicer arietinum*]) crops. In addition, the availability of these resources has facilitated their use in non-model crops leading to analysis of gene families, gene discovery, differential gene expression of various genes/gene families specific to stress responses, plant growth and development. To provide a comprehensive overview, the manuscripts in this Research Topic are categorized and summarized below under various sub-headings.

## Marker development, germplasm characterization, and genome mapping/gene discovery/gene analysis

Discovery of molecular markers followed by their use in crop improvement programs is one of the most important activities in crop genomics/molecular breeding. A new class of molecular markers termed “micro-RNA derived SSR markers” has been developed and used by Sihag et al. in characterizing a set of wheat genotypes for important trait “heat tolerance”. The study led to the identification of 104 heat-stress-responsive miRNAs already reported in different crop plants and validated a set of 70 miRNA-SSRs on 20 contrasting genotypes for heat tolerance. However, among these miRNA-derived SSRs markers, a set of only 19 were found polymorphic. These polymorphic markers were further tried for the study of genetic diversity and population structure of the selected wheat genotypes for heat tolerance. The analysis of genotypic data revealed that a smaller number of alleles (2.9 alleles per locus) were detected. Further analysis identified two important markers “miR159c and miR165b” that could discriminate between contrasting genotypes declared as promising diagnostic markers. We believe the results of the study are very important and may help in characterization of wheat germplasm for heat tolerance at the earliest stage and thus can save lot of time, efforts and money. The markers could be also used in wheat molecular breeding programs through MAS.

In addition to marker discovery, availability of all the marker resources developed for important traits in different crops in a single database would provide a useful catalog for marker assisted breeding activities. In this direction, Kumar S. et al. integrated, analyzed, and characterized the ESTs responsible for important abiotic stresses in some important cereals in the database “CerealESTdb”. The data base will prove useful for scientists working in the area. The data base can prove useful to fetch the information about ESTs from important crops like rice, wheat, sorghum, and maize. The data base possess a set of 55,826 assembled EST sequences. In addition, a set of 51,791 predicted genes models and 254,609 gene ontology terms including extensive information on 1,746 associated metabolic pathways can be accessed in the data base. More efforts need to be made to help decipher complex biological phenomena under abiotic stresses. The “CerealESTdb” database can be publicly accessed at “URL http://cabgrid.res.in/CerealESTDb”.

In one of the most important studies genetics-based senescence in maize, Caidedo et al. carried out GWAS to discover causative genetic variants influencing the major physiological measures of senescence. Plants use senescence as a defense mechanism against abiotic and biotic stresses. In this study, four physiological and two agronomic traits influencing senescence were recorded. The authors evaluated a set of 672 recombinant inbred lines (RILs) for two consecutive years. A set of 36 candidate genes were identified, including 11 genes supported by additional evidence for their involvement in senescence-related processes. Further, involvement of a key determinant of senescence “Zm00001d043586” in chlorophyll rate degradation was identified during late plant development stages. This study enhances our understanding of the genetic relationship between senescence and physiology related parameters in maize and provides new putative molecular markers for use in marker assisted selection.

The characterization of crop germplasm is one of the crucial steps in crop improvement programs. In this direction, an article by Hussain et al. presented characterization of Pakistani bread wheat germplasm Research Topic using genotyping by sequencing (GBS) approach. The study used a diverse germplasm of 184 Pakistani wheat genotypes. The genotypes were subjected to high-throughput genotyping (GBS genotyping) with a set of 123,596 high-quality single nucleotide polymorphism (SNP) markers. The study presented a detailed distribution of SNPs on different bread wheat genomes and several genetic diversity parameters along with estimates of genome-wide linkage disequilibrium. The results of population structure, principal coordinate analysis, phylogenetic tree, and kinship analysis will prove useful in future Pakistani wheat breeding programs and in the study of marker-trait associations for important traits. Similarly, Brhane et al. used GBS based SNP markers for the study of genetic diversity in finger millet**.** The GBS method was used to simultaneously identify SNP markers as well as to genotype 288 finger millet genotypes collected from Ethiopia (228 landraces and 14 cultivars) and Zimbabwe (46 landraces). The 288 genotypes were further structured into seven sub-populations. Further analysis through AMOVA revealed genetic differentiation among sub-populations classified on the basis of geographical origin and trait data. The resultant genetic diversity from this study has the potential to improve future breeding programs for finger millet.

Leaf rust is one of the most devastating fungal diseases affecting wheat globally. More than 80 genes/QTLs have been identified for this disease on different chromosomes. In addition, some novel genes have also been identified for this disease. Kumar K. et al. presented a very comprehensive and timely review on leaf rust resistant genes and the development of resistant varieties. The review provided particularly valuable information on all of the leaf rust resistance genes including novel genes identified from different studies. Leaf rust resistance genes identified through QTL interval mapping and GWAS have been summarized in the review. The leaf rust resistance genes introgression/pyramiding into commercial/prominent cultivars from 18 different countries including India have been also tabulated. A comprehensive presentation of the challenges and future perspectives for breeding of leaf rust resistance will prove useful for global programs in wheat genetics and breeding. Another article in this Research Topic by Bhurta et al. demonstrated the importance of genome-wide identification and expression analysis of the Thioredoxin (*Trx*) gene family in wheat and examined the implication of *Trx* genes in leaf rust resistance. Thioredoxin (Trx) is a low molecular weight, thermostable, redox regulator. Redox regulators can control cellularhomeostasis viareactive oxygen species (ROS) and reactive nitrogen species (RNS) in response to pathogenic infections. The study identified a total of forty-two (42) wheat *Trx* genes (*TaTrx*) across the wheat genomes, with 12 on A, 16 on B, and 14 on D genomes, respectively. Further, eight of the 15 *TaTrx* genes selected based on *in silico* expression analysis, were further confirmed to be differentially targeted by microRNAs (miRNA). Gene expression analysis usingquantitative real-time PCR (qRT-PCR) revealed that *TaTrx* genes, *TaTrx11-5A*, *TaTrx13-5B*, *TaTrx14-5D*, and *TaTrx15-3B* are significantly induced in response to leaf rust infection in wheat (Bhurta et al.).

## Functional genomics/transcriptomics

In plants, several studies reported an important role of transcription factor (TF) gene families in diverse processes such as developmental control and elicitation of defense/stress responses. For example, in rice, genome wide analysis of dehydrin (DHNs) gene family revealed that *DHNs* play an important role in combating dehydration stress (Verma et al., 2017). Similarly, the identification of AP2/ERF TF superfamily genes in five legumes *Medicago*, lotus, common bean, chickpea and pigeon-pea along with expression profiling in chickpea and pigeon-pea uncovered implications for *AP2/ERF* and *HSP90* genes in biotic and abiotic stresses (Agarwal et al., 2016). In this Research Topic, several articles are devoted to the significant role of functional genomics in the identification of gene families that control plant growth, development, biotic/abiotic stresses. For instance, the MADS-box gene family members serve a variety of roles in crop plant growth and development, and they hold a lot of promise for increasing grain yields under changing global conditions. An article in this issue by Shao et al. focused on the identification and expression analysis of MADS-Box gene family in sweet potato [*Ipomoea batatas* (L.) Lam]. MADS-box is one of the largest TF families in plants, with important roles in plant growth and development. This study provided a comprehensive analysis and identification of 95 MADS-box genes in sweet potato. These genes were analyzed and further categorized based on phylogenetic analysis with *Arabidopsis* MADS-box proteins. The MADS domain and core motifs of the sweet potato MADS-box genes were identified, with 19 genes showing collinear relationships and duplication events. Furthermore, RNA-Seq data suggested tissue-specific expression patterns for MADS box genes, including 34 being highly expressed in flowers and fruits, and 19 being highly expressed in the roots. Validation of these expressed genes though qRT-PCR revealed higher expression of five genes, *viz.* *IbMADS1*, *IbMADS18*, *IbMADS19*, *IbMADS79*, and *IbMADS90* in the tuberous root or fibrous root, and three genes *IbMADS18*, *IbMADS31*, and *IbMADS83* in the fruit. This study investigated the effects of MADS-box genes on the growth and development of sweet potato which provided molecular clues for use in sweet potato breeding.

In another article, the limited knowledge on MADS-box genes in the wheat genome is being rectified by a genome-wide investigation of the publicly accessible wheat reference genome undertaken by Raza et al. and has led to the identification of 300 MADS-box genes with high confidence. The identified genes were classified into 16 subfamilies after phylogenetic analysis with *Arabidopsis* and rice MADS-box genes. The *in silico* expression data suggested that MADS-box genes function as active plant protectors against pathogen insurgency and harsh climatic circumstances. The authors further presented a comprehensive set of MADS-box genes in the wheat genome, which could help speed up functional genomics studies and bridge gaps between genotype-to-phenotype correlations by fine-tuning agronomically relevant features.

High-throughput sequencing technologies have been widely used to generate large amounts of genome-wide gene expression data (Kudapa et al., 2018). Gene expression patterns aid in better understanding of the functional elements of the genome, and also enable insights into development and disease resistance in plants. In addition to transcriptome analysis in a single genotype or tissue, comparative transcriptome analysis from multiple lines/growth stages/tissues can demonstrate numerous essential biochemical pathways and common genes in response to one or more stresses. Several prior studies have validated the effectiveness of comparative transcriptomics. For example, in wheat, comparative transcriptomics analysis was performed in susceptible and resistant wheat lines to identify genes and pathways that are targeted by obligate biotrophic fungal pathogens (Poretti et al., 2021). Similarly, in chickpea, comparative transcriptome analysis of control and inoculated roots of three resistant/susceptible genotypes revealed key genes associated with root-lesion nematode resistance (Channale et al., 2021).

In the current issue, comparative transcriptomic analysis was conducted in the Russian dandelion (*Taraxacum koksaghyz*) between plant populations differening in their root morphology (sizes and shapes), to identify key genetic determinants of root morphology (Wieghaus et al.). The genetics underlying the size and shape of roots of the Russian dandelion are key tragets for improvement as it is the primary storage location for natural rubber and inulin. Further, the Russian dandelion is considered an alternative source of natural rubber to the natural rubber tree (*Hevea brasiliensis*). The study identified differentially expressed genes correlating with different root morphotypes, resulting in the identification of multiple candidate genes, including a *glucan endo-1,3-β-d-glucosidase*, an *allene oxide synthase 3*, a TIFY10A/JAZ1 homolog. The results were further validated using qRT-PCR for two genes in Russian dandelion plants and its correlation with different root morphologies. These results confirm the effectiveness of comparative transcriptomics and its deployment in marker-assisted breeding of the Russian dandelion (Wieghaus et al.).

Our special topic also featured the elucidation of gene family research in crops, *Perilla frutescens* and *Euscaphis konishii* known for their medicinal values in Asia. Both studies were facilitated by the availability of publicly available genome and transcriptomic datasets of these species. Duan et al. conducted genome-wide analysis of the fatty acid desaturase (*FAD*) gene family in *Perilla* (*P. frutescens*) seeds known for their high α-Linolenic Acid (ALA) content and characterized 42 members of the *FAD* gene family which are known to be important players in the ALA biosynthesis pathway. This study also performed bioinformatic analyses of conserved domains, cis-regulatory elements, phylogenetic analyses, and characterized gene expression profiles in various tissue types. Further characterization of a *Perilla FAD* gene using overexpression in *Arabidopsis thaliana* demonstrated anincrease in ALA content in seeds.

The second study conducted by Liu et al. focused on characterization of 27 Auxin Response Factors (ARF) and 34 Auxin/Indole-3-acetic acid (Aux/IAA) protein families thus providing better understanding of their role in triterpenoid and anthocyanin biosynthesis in *Euscaphis konishii* fruits. This study also provides bioinformatic analyses of conserved domains, cis-regulatory elements, phylogenetic analyses and gene expression profiles characterized in various tissue types. The analysis of results suggested that *EkIAA* and *EkARF* genes remained conserved. Further, downstream analyses using qRT-PCR, yeast one-hybrid, and transient expression provide insight into the interaction between *EkARF5.1* and auxin response elements. This study provides gene targets including *EkARF5.1*that could help with the objective of crop improvement.

Under adverse conditions, superoxide dismutase (SOD) enzymes acts as the first line of defense in the plant antioxidant system, removing reactive oxygen species (ROS). SOD proteins are found throughout the plant kingdom and serve an important role in plant growth and development. Cucurbitaceae, the gourd family consisting of about 975 species is a significant economic and nutritional crop in the world. However, this crop has not undergone a full investigation of the *SOD* gene family. Rehman et al. conducted a genome-wide comparative analysis of the *SOD* gene family in Cucurbitaceae as well as gene expression analysis of *Lagenaria siceraria* under different abiotic stresses. The study identified 43 *SOD* genes from Cucurbitaceae species and analysis of these genes revealed that *SOD* genes could be divided into eight subfamilies. Further, the structural analysis of *SOD* gene family shows that the exon/intron structure and motifs are well preserved. Further, phylogenetic and population structure analysis revealed functional divergence of the Cucurbitaceae *SOD* gene family. Also, qRT-PCR of eight *Lsi SOD* genes shows differential response to various abiotic stresses, including droughtand cold stress. This study laid the groundwork for further detailed analysis of the role of the *SOD* gene family in Cucurbitaceae for improvement of this important crop.

Similarly, Wen et al. performed genome-wide analysis of *TCP* genes in the rapeseed genome, which included*in silico* characterization and gene expression analysis. The study led to the identification of 80 *BnTCP* genes and these genes could be grouped into two main classes (Class I - *PCF* and Class II - *CYC/TB1*) based on phylogenetic analysis. Further gene structural analysis shows diverse intron exon patterns as well as number of motifs, and phylogentic analysis shows a pattern of positive selection and duplications. The study also identified four hormone- and four stress-related responsive *cis*-elements in the promoter regions. Gene ontology enrichment analysis, shows enrichment within RNA/DNA binding, metabolic processes, and transcriptional regulatory activities. Transcriptome widetissue-specific expression analyses indicates that only a few genes (*BnTCP9*, *BnTCP22, BnTCP25, BnTCP48, BnTCP52, BnTCP60, BnTCP66* and *BnTCP74*) are upregulated in root, stem, leaf, flower, seeds, and silique. Similarly, qRT-PCR-based expression analysis indicates that *BnTCP36, BnTCP39, BnTCP53, BnTCP59* and *BnTCP60* are upregulated at certain timepoints uponhormone treatment and abiotic stresses. These genes did not respond to drought and methyl jasmonate (MeJA). These insights pave the way for future research into importance of *BnTCP* gene family in rapeseed, particularly for tolerance to abiotic stresses.

Ren et al. studied the expression and association analysis of *CBLL* genes in peanut. In the study, *CBL*-like genes were classified as those with the Cys Met Meta PP domain. A set of 29 *CBLL* genes including 12 from cultivated peanuts and 17 from wild species were identified. The genes were found scattered at the ends of different chromosomes. These identified *CBLL* proteins were found to have five separate evolutionary branches based on evolution, gene structure, and motif analysis. The chromosome distribution patterns and synteny analyses identified whole-genome duplication (allo-polyploidization) leading to the expansion of *CBLL* genes. Further comparative genomics investigation showed that there were three shared collinear *CBLL* gene pairs between peanut, *Arabidopsis*, grape, and soybean, but none between peanut and rice. The authors also identified one polymorphic site in *AiCBLL7* through association analysis. This site is believed to be closely associated with pod length (PL), pod width (PW), hundred pod weight (HPW) and hundred seed weight (HSW).

Hong et al. performed a comparative transcriptomics study under different abiotic stresses, to better understand the role of *A. thaliana* gene *AtARA6* and its contribution to abiotic stress tolerance in soybean. The gene is known to play an important role in salt tolerance in *A. thaliana*. Transcriptome sequencing and subsequent differential expression analysis of transgenic soybean overexpressing *AtARA6* and wild-type, “Shen Nong 9” (SN9) under salt treatment and water treatment indicate several key categories of genes, including transcription factors such as WRKY86 that are significantly impacted after salt treatment. The results suggest the role of *AtARA6* gene in salt tolerance in transgenic soybean *via* regulation of soybean SNARE complexes. Thus, these investigations provide the gene expression changes mediated by abiotic stresses in transgenic soybean overexpressing *AtARA6* gene, and some insights into the mechanism mediating salt tolerance in soybean.

## Genome editing and omics approaches for functional foods

Functional foods are plant-based foods that provide health advantages in addition to basic nourishment, such as fruits, vegetables, spices, cereals, millets, pulses, and oilseeds. Few crops have been studied to uncover their use as functional foods. Nayak et al. compiled a very important review article on recent advances made in different omics tools and technologies for enhancing plant based functional foods. A variety of omics approaches/integrated omics including genomics, transcriptomics, proteomics, metabolomics coupled with artificial intelligence and machine learning approaches have been proposed to improve crops plant yields and/or functional profiles. The authors have been made to compile literature and provide comprehensive insights into omics studies that have been carried out to uncover active components and crop improvement by enhancing the functional compounds in different crop plants. Further efforts are required to characterize functional foods used in traditional medicines, as well as application of this knowledge to improve biofortification of staple food crops in order to tackle malnutrition and hunger in the world.

Among the several recently emerging genomics tools and techniques, CRISPR-Cas is one of the most promising genome editing tools being used in crop improvement programs. Therefore, a comprehensive review was presented by Joo et al. on “Utilization of CRISPR-Cas in Tropical Crop Improvement”. The authors provided a clear workflow with distinctprocesses with specific steps as well as decision points that should make practical application of the CRISPR-Cas gene editing platform more structured. The authors outlined different steps involved in the process with the aim of democratizing the technology and helping any lab planning to implement CRISPR-cas in their workflow.

## Perspective

The contributions in this Research Topic provide a wealth of information on different aspects of genetics, genomics, crop biotechnology, and molecular plant biology. Genomic technologies, procedures, and statistical methodologies must continue to evolve in order to advance the discipline and breed genotypes that will fulfill future food needs as well as adaptability of crops to climate change. The development as well as use of new marker types derived from different sources, such as miRNA-derived SSRs, are essential for examining genetic linkages, population structure analyses, and marker-trait interactions with different important traits. Furthermore, developments in NGS technology have made it easier to construct and use pangenomes which will expand the number of DNA markers available for GWAS. These marker resources will aid in utilizing hitherto untapped genetic resources to accurately breed for the desired characteristic in a short period of time, which would have been otherwise difficult to achieve.

The utilization of high-throughput genotyping systems for the study of genetic diversity, population structure, functional genomics, transcriptomics, whole-genome scanning and mapping genes/QTLs for critical features like biotic/abiotic stress tolerance, and climate resilience, were underscored in several publications in the Research Topic. Emphasis was also laid on genome-wide analysis of different gene families in important crop plants and in non-model crop plants. These gene families are responsible for governing important traits. Bi-parental mapping populations for QTL/gene discovery and varied germplasm resources for genome-wide association studies (GWAS) were utilized. However, study and analyses of multi-parental mapping populations can improve precision gene/QTL identification. Furthermore, new advanced gene discovery methods/multi-locus approaches are needed to uncover genes/QTLs, which can lead to the discovery of candidate genes for functional characterization by gene deletion, mutagenesis, and genome editing. The Research Topic aims to demonstrate how to use multi-parental populations and QTL identification to breed genotypes for different traits and encourages readers to apply this knowledge to breeding next-generation crop varieties.

## References

[B2] Banerjee S. Das S. Mir R. R. Kundu A. Topdar N. Sarkar D. (2012). Assessment of genetic diversity and population structure in a selected germplasm collection of 292 jute genotypes by microsatellite (SSR) markers. Mol. Plant Breed. 3, 11–25. 10.5376/mpb.2012.03.0002

[B3] Barmukh R. Roorkiwal M. Garg V. Khan A. W. German L. Jaganathan D. (2022). Genetic variation in CaTIFY4b contributes to drought adaptation in chickpea. Plant Biotechnol. J. 20, 1701–1715. 10.1111/pbi.13840 35534989 PMC9398337

[B4] Channale S. Kalavikatte D. Thompson J. P. Kudapa H. Bajaj P. Varshney R. K. (2021). Transcriptome analysis reveals key genes associated with root-lesion nematode *Pratylenchus thornei* resistance in chickpea. Sci. Rep. 11, 17491. 10.1038/s41598-021-96906-3 34471168 PMC8410808

[B5] Choudhary N. Anjali G. M. Shafi S. Jan S. Mir A. Singh B. (2021). Molecular diversity and nutriment studies of common bean (Phaseolus vulgaris L.) from the two hot-spots of western himalayas of Jammu and Kashmir. Crop Pasture Sci. 73, 249–262. 10.1071/CP21347

[B6] Gupta P. K. Kulwal P. L. Mir R. R. (2013b). “QTL mapping: Methodology and applications in cereal breeding,” in Cereal genomics II. Editors K Gupta P. K Varshney R. (Netherlands: Springer). 10.1007/978-94-007-6401-9_11

[B7] Gupta P. K. Rustgi S. Mir R. R. (2013a). “Array-based high-throughput dna markers and genotyping platforms for cereal genetics and genomics,” in Cereal genomics II. Editors Gupta P. K. Varshney R. K. (Netherlands: Springer). 10.1007/978-94-007-6401-9_2

[B8] Gupta P. K. Rustgi S. Mir R. R. (2008). Array-based high-throughput DNA markers for crop improvement. Heredity 101, 5–18. 10.1038/hdy.2008.35 18461083

[B9] Jamaloddin M. Durga Rani C. V. Swathi G. Anuradha C. Vanisri S. Rajan C. P. (2020). Marker Assisted Gene Pyramiding (MAGP) for bacterial blight and blast resistance into mega rice variety “Tellahamsa”. PloS one 15 (6), e0234088. 10.1371/journal.pone.0234088 32559183 PMC7304612

[B10] Khan A. W. Garg V. Roorkiwal M. Golicz A. A. Edwards D. Varshney R. K. (2020). Super-pangenome by integrating the wild side of a species for accelerated crop improvement. Trends Plant Sci. 25 (2), 148–158. 10.1016/j.tplants.2019.10.012 31787539 PMC6988109

[B11] Kudapa H. Garg V. Chitikineni A. Varshney R. K. (2018). The RNA‐Seq‐based high resolution gene expression atlas of chickpea (*Cicer arietinum* L.) reveals dynamic spatio‐temporal changes associated with growth and development. Plant Cell Environ. 41 (9), 2209–2225. 10.1111/pce.13210 29637575

[B12] Kulwal P. L. Mir R. R. Varshney R. K. (2022). “Efficient breeding of crop plants,” in Fundamentals of field crop breeding. Editor Yadav D. K. (Singapore: Springer). 10.1007/978-981-16-9257-4_14

[B13] Kumar S. Kumar M. Mir R. R. Kumar R. Kumar S. (2021). “Advances in molecular markers and their use in genetic improvement of wheat,” in Physiological, molecular, and genetic perspectives of wheat improvement. Editors Wani S. H. Mohan A. Singh G. P. (Springer), 139–174.

[B14] Mir R. R. Choudhary N. Singh B. Khandy I. Bawa V. Sofi P. (2015). “Harnessing genomics through phenomics,” in Phenomics in crop plants: Trends, options and limitations. Editor Kumar J. (India: Springer).

[B15] Mir R. R. Reynolds M. Pinto F. Khan M. A. Bhat M. A. (2019). High-throughput phenotyping for crop improvement in the genomics era. Plant Sci. 282, 60–72. 10.1016/j.plantsci.2019.01.007 31003612

[B16] Mir R. R. Rustgi S. Zhang Y-M. Xu C. (2022). Multi-faceted approaches for breeding nutrient-dense, disease resistant, and climate resilient crop varieties for food and nutritional security. Heredity 128, 387–390. 10.1038/s41437-022-00542-0 35606571 PMC9177835

[B17] Mir R. R. Saxena R. K. Saxena K. B. Upadhyaya H. D. Kilian A. Cook D. R. (2013b). Whole-genome scanning for mapping determinacy in pigeonpea (*Cajanus* spp.). Plant Breed. 132, 472–478. 10.1111/j.1439-0523.2012.02009.x

[B18] Mir R. R. Hiremath P. J. Riera-Lizarazu O. Varshney R. K. (2013a). “Evolving molecular marker technologies in plants: From RFLPs to GBS,” in Diagnostics in plant breeding. Editors Lübberstedt T. Varshney R. K. (New York: Springer Science+Business), 229–247.

[B19] Mir R. R. Varshney R. K. (2013). “Future prospects of molecular markers in plants,” in Molecular markers in plants. Editor Henry R. J. (Oxford, UK: Blackwell Publishing Ltd), 169–190.

[B20] Mir R. R. Zaman-Allah M. Sreenivasulu N. Trethowan R. &Varshney R. K. (2012). Integrated genomics, physiology and breeding approaches for improving drought tolerance in crops. Theor. Appl. Genet. 125, 625–645. 10.1007/s00122-012-1904-9 22696006 PMC3405239

[B22] Pazhamala L. T. Kudapa H. Weckwerth W. Millar A. H. Varshney R. K. (2021). Systems biology for crop improvement. Plant Genome 14, e20098. 10.1002/tpg2.20098 33949787 PMC12806876

[B23] Poretti M. Sotiropoulos A. G. Graf J. Jung E. Bourras S. Krattinger S. G. (2021). Comparative transcriptome analysis of wheat lines in the field reveals multiple essential biochemical pathways suppressed by obligate pathogens. Front. Plant Sci. 12, 720462. 10.3389/fpls.2021.720462 34659291 PMC8513673

[B24] Raza A. Tabassum J. Kudapa H. Varshney R. K. Sagwal V. Sihag P. (2021). Can omics deliver temperature resilient ready-to-grow crops?Development and characterization of nitrogen and phosphorus use efficiency responsive genic and miRNA derived SSR markers in wheat. HeredityHeredity 41 (8), 391–401. 10.1038/s41437-022-00506-4 PMC9177559 35132208

[B25] Sagwal V. Sihag P. Singh Y. Mehla S. Kapoor P. Balyan P. (2022). Development and characterization of nitrogen and phosphorus use efficiency responsive genic and miRNA derived SSR markers in wheat. Hered. Nat. 128, 391–401. 10.1038/s41437-022-00506-4 PMC9177559 35132208

[B26] Shafi S. Tahir M. Khan M. A. Bhat M. A. Kumar U. Kumar S. (2021). Trait phenotyping and SSR markers characterization of wheat (*Triticum aestivum* L.) germplasm for breeding early maturing wheat’s for Western-Himalayas. Genet. Resour. Crop Evol. 69, 755–770. 10.1007/s10722-021-01261-x

[B27] Tahir M. Shafi S. Khan M. A. Sheikh F. A. Bhat M. A. Sofi P. A. (2021). Grain micronutrient and molecular characterization of wheat (*Triticum aestivum* L.) germplasm using genic and random SSR Markers. Crop Pasture Sci. 73, 93–103. 10.1071/CP21116

[B28] Tyagi S. Kumar A. Gautam T. Pandey R. Rustgi S. Mir R. R. (2021). Development and use of miRNA-derived SSR markers for the study of genetic diversity, population structure, and characterization of genotypes for breeding heat tolerant wheat varieties. PLoS One 16 (2), e0231063. 10.1371/journal.pone.0231063 33539339 PMC7861453

[B29] Tyagi S. Sharma S. Ganie S. A. Tahir M. Mir R. R. Pandey R. (2019). PlantmicroRNAs: Biogenesis, gene silencing, web-based analysis tools and their use as molecular markers. 3 Biotech. 9 (11), 413. 10.1007/s13205-019-1942-y PMC6811466 31696018

[B30] Verma G. Dhar Y. V. Srivastava D. Kidwai M. Chauhan P. S. Bag S. K. (2017). Genome-wide analysis of rice dehydrin gene family: Its evolutionary conservedness and expression pattern in response to PEG induced dehydration stress. PLoS One 12 (5), 0176399. 10.1371/journal.pone.0176399 PMC5411031 28459834

